# Enhanced Recovery After Surgery for Breast Reconstruction: Pooled Meta-Analysis of 10 Observational Studies Involving 1,838 Patients

**DOI:** 10.3389/fonc.2019.00675

**Published:** 2019-07-30

**Authors:** Ya-Zhen Tan, Xuan Lu, Jie Luo, Zhen-Dong Huang, Qi-Feng Deng, Xian-Feng Shen, Chao Zhang, Guang-Ling Guo

**Affiliations:** ^1^Center of Women's Health Sciences, Taihe Hospital, Hubei University of Medicine, Shiyan, China; ^2^Center for Evidence-Based Medicine and Clinical Research, Taihe Hospital, Hubei University of Medicine, Shiyan, China; ^3^Department of General Surgery, Taihe Hospital, Hubei University of Medicine, Shiyan, China

**Keywords:** breast reconstruction, enhanced recovery after surgery, pain control, flap loss, complication

## Abstract

**Purpose:** This study aims to explore the effectiveness and safety of the enhanced recovery after surgery (ERAS) protocol vs. traditional perioperative care programs for breast reconstruction.

**Methods:** Three electronic databases (PubMed, EMBASE, and Cochrane Library) were searched for observational studies comparing an ERAS program with a traditional perioperative care program from database inception to 5 May 2018. Two reviewers independently screened the literature according to the inclusion and exclusion criteria, extracted the data, and evaluated study quality using the Newcastle-Ottawa Scale. Subgroup and sensitivity analyses were performed. The outcomes included the length of hospital stay (LOS), complication rates, pain control, costs, emergency department visits, hospital readmission, and unplanned reoperation.

**Results:** Ten studies were included in the meta-analysis. Compared with a conventional program, ERAS was associated with significantly decreased LOS, morphine administration (including postoperative patient-controlled analgesia usage rate and duration; intravenous morphine administration on postoperative day [POD] 0, 1, 2, and 4; total intravenous morphine administration on POD 0–3; oral morphine consumption on POD 0–4; and total postoperative oral morphine consumption), and pain scores (postoperative pain score on POD 0 and total pain score on POD 0–3). The other variables did not differ significantly.

**Conclusion:** Our results suggest that ERAS protocols can decrease LOS and morphine equivalent dosing; therefore, further larger, and better-quality studies that report on bleeding amount and patient satisfaction are needed to validate our findings.

## Introduction

Breast cancer is the most common cancer diagnosis in women, with 30–40% of patients undergoing mastectomy as treatment (1). Long-term quality of life and cosmetic outcomes after different methods are important considerations for patients that choose breast cancer treatment (2). Research shows that breast reconstruction following surgical treatment for breast cancer improves patient satisfaction and health care-related quality of life (3). Thus, in the United States, breast reconstruction is considered as a standard part of care for breast cancer patients treated with mastectomy (4), with a 39% increase in procedural volume since 2000 (5). However, in most cases, the length of hospital stay (LOS) increases and postoperative complications remains a challenge for patients who have undergone breast reconstruction (6).

Emerging evidence suggests that one effective strategy for reducing postoperative complications may be the adoption of an enhanced recovery after surgery (ERAS) program that uses a transdisciplinary comprehensive approach to perioperative care (7). ERAS is a collective, standardized, evidence-based preoperative, intraoperative, and postoperative multidisciplinary protocol involving the collaboration of several specialties and focuses on engaging patients and their families in their care and ensuring that uniform evidence-based bundled care is delivered with the primary goal of reducing the LOS (1). In the current health care environment, hospitals must achieve a delicate balance between limiting expenses and delivering high-quality care (8). Using evidence-based models, clinicians have successfully tested ERAS protocols to deliver comprehensive perioperative care that is patient-centered and efficient and reduces variations in outcomes such as LOS (9). The important elements of ERAS and similar fast-track surgery (FTS) programs in breast reconstructive surgery included in these studies were factors that improved outcomes; many also addressed traditional outdated treatments. These measures were then amalgamated into treatment programs that included preoperative carbohydrate loading, postoperative nausea and vomiting prophylaxis, and other methods (10).

One systematic review of breast reconstruction published in 2016 also analyzed LOS and postoperative complications (11); in this article, the number of studies included was inadequate at only three. Another study of microsurgical breast reconstruction published in 2017 was the minutes taken during a meeting (12). The third study, published in 2018, included nine systematic reviews and meta-analyses of breast reconstructions (13). Therefore, here we included more studies to confirm our results through detailed systematic reviews and meta-analyses. We conducted a comprehensive and systematic analysis of postoperative complications and added research on pain control and readmission. ERAS protocols have also been implemented in breast reconstruction surgery, but their effectiveness has not been studied extensively. We therefore performed a pooled analysis to investigate the effect of ERAS/FTS pathways compared to conventional programs on decreasing LOS, reducing postoperative complication and readmission rates, and relieving pain.

## Methods

### Search Strategy

We systematically searched the PubMed, EMBASE, and Cochrane Library databases from their inception to 5 May 2018. Publication language was restricted to English. Detailed search strategies are shown in Supplemental Method 1.

### Inclusion and Exclusion Criteria

Studies were considered eligible for inclusion if they met all of following inclusion criteria: (1) Adult patients undergoing breast reconstruction surgery; (2) Perioperative care using ERAS or FTS protocols vs. standard or conventional care; (3) Reported outcomes including at least LOS, complication rates, pain control, emergency department visits, hospital readmission, and unplanned reoperation and costs; and (4) Full-text cohort and case-controlled studies published in English.

A study was excluded if: (1) It did not compare ERAS with a traditional method; (2) Its original research data could not be used, and the consulted authors had not obtained useful results; and (3) It examined aesthetic procedures or mastectomy alone.

### Data Extraction and Quality Assessment

Two authors screened the abstracts and titles of the studies identified in the initial search, and independently read the full text of the selected studies. Disagreements were resolved by a third researcher. The data were extracted independently by two authors.

The methodological quality of the included cohort or case-cohort studies was assessed independently by two commentators using the Newcastle-Ottawa Scale (NOS). Studies that achieve six or more stars on the modified NOS were considered high quality (14).

### Statistical Analysis

For continuous outcome data, means, and standard deviations were used to calculate mean differences (MD) in the meta-analysis (15); for dichotomous outcomes, relative risk (RR) was calculated (16). Each effect amount gives a 95% confidence interval (CI). Initial analyses were performed using a fixed-effects model. Statistical heterogeneity was tested using *I*^2^ tests (17), which provides an estimate of the percentage of inconsistency thought to be due to chance (18). We determined the use of the model based on the *I*^2^ value, most of which are considered *I*^2^ >40% and using a random effects model when *I*^2^ ≤ 40%. The level of significance for all tests, including heterogeneous statistics, was set at an alpha level of 0.05. A subgroup analysis was performed of certain factors that may affect overall outcomes, including pain management, hospitalization LOS, and complications. We performed a sensitivity analysis of article types, analyzed the data, and reported the results through relevant experiments. All statistical analyses were performed using R software.

## Results

### Literature Identification

**Figure 1:**
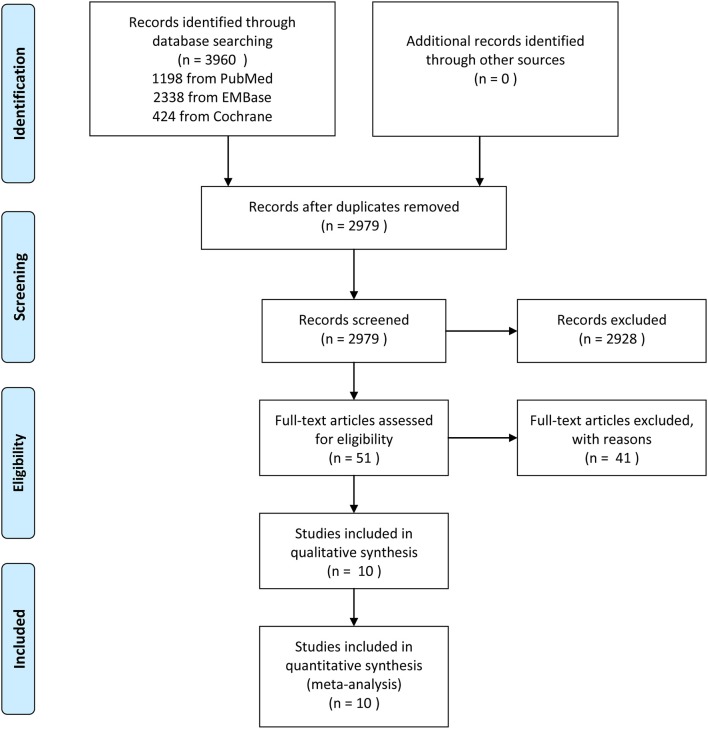
Outline of screening and identification of studies.

In the initial literature search, 3,960 studies were identified. After the removal of 981 duplicate studies, 2,979 potentially relevant studies were screened on the basis of citations, of which 2,928 were excluded because they did not meet the inclusion criteria, leading to the evaluation of 51 full texts. Forty-two studies were removed after careful full-text screening; the specific reasons for exclusion are recorded in detail (Supplemental Table 1). Ultimately, 10 studies were included in the meta-analysis (Figure 1).

### Study Characteristics, ERAS Elements, and Quality Evaluation

Ten studies (1, 5, 6, 8, 19–24) included in the review were published between 2015 and 2018, including eight after autologous breast reconstruction surgery and two after implant-based breast reconstruction surgery. Aside from one case-control study, the studies were cohort studies (Table 1).

**Table 1:** Patients' and studies' characteristics.

**References**	**Age (T/E)**	**Study design**	**Surgery type**	**Sample**		**Unilateral (T/E)**	**Bilateral (T/E)**
				**T**	**E**		
Afonso et al. (6)	51/50	Cohort study	Immediate or delayed	49	42	29/21	20/21
Astanehe et al. (19)	50.2/52.7	Cohort study	Immediate or delayed	169	72	64/27	105/45
Batdorf et al. (8)	47.5/48.3	Cohort study	Immediate or delayed	51	49	10/9	41/40
Bonde et al. (20)	51/53.9	Case control study	NA	277	177	277/177	0/0
Chiu et al. (1)	48.8/46.9	Cohort study	Immediate or delayed	276	96	111/40	165/56
Dumestre et al. (21)	49/45	Cohort study	Immediate and delayed	78	78	15/35	63/43
Dumestre et al. (22)	48/48	Cohort study	Immediate and delayed	29	29	11/5	18/24
Kaoutzanis et al. (5)	51/51.9	Cohort study	Immediate and delayed	50	50	27/28	23/22
Oh et al. (24)	49.4/49.2	Cohort study	Immediate and delayed	118	82	32/10	86/72
Odom et al. (23)	49.0/49.8	Cohort study	Immediate and delayed	47	19	21/7	26/12

*TRAS, Traditional recovery after surgery; ERAS, Enhanced recovery after surgery; T, TRAS; E, ERAS; NA, Not applicable*.

ERAS elements used a consensus review (10) in 2017, with a total of 18 recommended items. A mean of nine (range, 4–12) ERAS elements were clearly shown for each ERAS protocol. Details of the ERAS protocols and conventional recovery regimens across the included studies are shown in Supplemental Table 2.

One case-control study and nine cohort studies were evaluated using the NOS. In eight of the cohort studies, the methods for determining exposure factors were reasonable and demonstrated that the outcomes of interest were not present at the start. In addition, the evaluation of the results was sufficient for all studies. Therefore, the number of stars in all studies was six or more. The case-control study also had six stars (Supplemental Table 3).

### Complications

#### Complications After Autologous Breast Reconstruction Surgery

**Figure 2:**
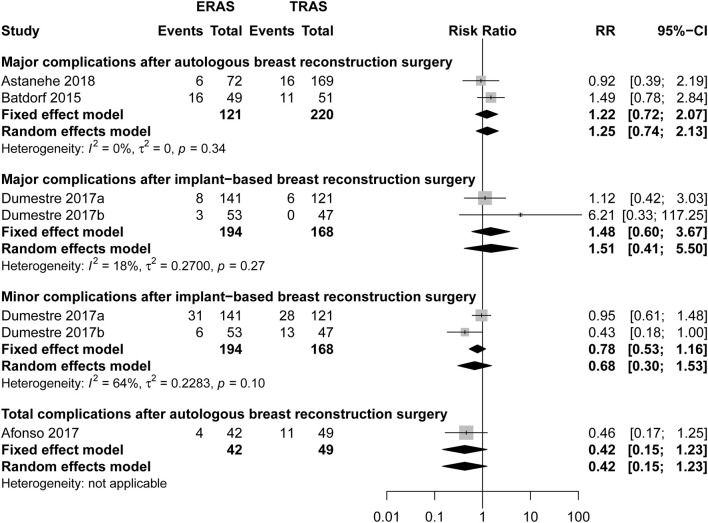
Pooled estimate of the effect of ERAS programs on incidence of total, major, and minor complications within 30 days after autologous and implant-based breast reconstruction surgery compared to conventional perioperative care programs. The incidence is based on number of breast reconstruction in Dumestre et al. (21) and Dumestre et al. (22).

There was no significant difference between ERAS/FTS and conventional programs in total or major (Figure 2; RR, 1.22; 95% CI, 0.72–2.07; *I*^2^ = 0%) complications within 30 days after surgery.

**Figure 3:**
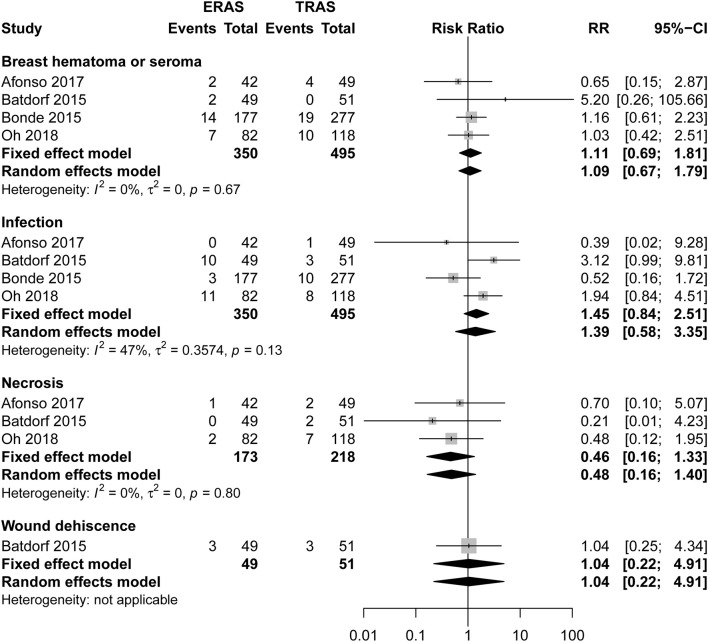
Pooled estimate of the effect of ERAS programs on incidence of breast-related complications within 30 days after autologous breast reconstruction surgery compared to conventional perioperative care programs.

**Figure 4:**
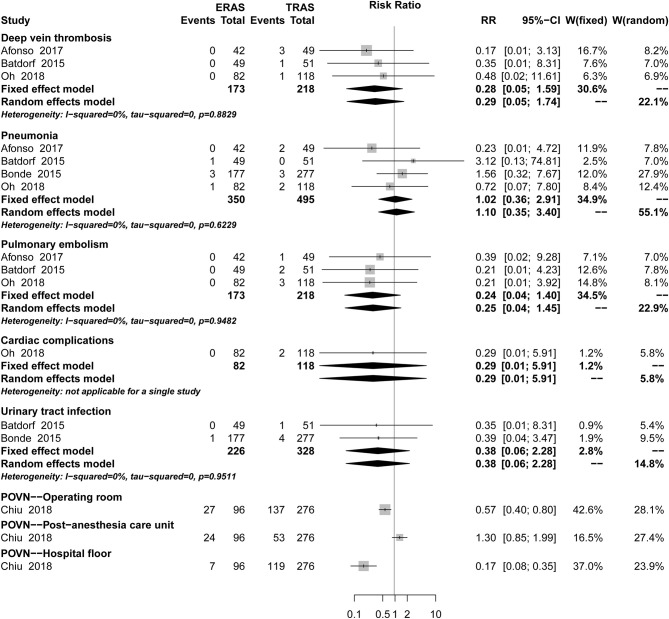
Pooled estimate of the effect of ERAS programs on incidence of systemic complications within 30 days after autologous breast reconstruction surgery compared to conventional perioperative care programs.

There was no significant difference between ERAS/FTS and conventional programs in the incidence of breast-related (Figure 3; Table 2), donor-site (Supplemental Figure 1), systemic (Figure 4), or opioid-related (Table 3; RR, 0.57; 95% CI, 0.28–1.16; *I*^2^ = 41%) complications and urinary tract infection (Figure 4; RR, 0.38; 95% CI, 0.06–2.28; *I*^2^ = 0%) within 30 days after surgery.

**Table 2:** Pooled estimate of the effect of ERAS programs on incidence of partial, total, and partial & total flap loss within 30 days after autologous and implant-based breast reconstruction surgery compared to conventional perioperative care programs.

**References**	**Number (ERAS/TRAS)**	**Flap type (ERAS/TRAS)**			**Partial flap loss**		**Total flap loss**		**Partial & Total flap loss (ERAS/TRAS)**
		**DIEP**	**MS-TRAM**	**TRAM**	**Definition**	**ERAS/TRAS**	**Definition**	**ERAS/TRAS**	
Afonso et al. (6)	42/49	28/28	14/16	0/5	NA	NA	NA	NA	1/0
Batdorf et al. (8)	49/51	60/39	25/44	4/9	<40% of the total flap (vascular compromise)	3/0	Complete loss of the flap due to microvascular arterial or venous thrombosis requiring explantation	2/1	5/1
Bonde et al. (20)	177/277	124/44	0/0	53/233	>5% of the total flap	7/9	NA	4/7	11/16
Oh et al. (24)	82/118	NA	NA	NA	NA	3/1	NA	2/1	5/2
Odom et al. (23)	19/47	15/40	NA	NA	NA	0/2	NA	2/1	2/3
Total	369/542	NA	NA	NA	13/12		10/10		24/22
RR (95%CI)	NA	NA	NA	NA	1.67 (0.77, 3.61)		1.55 (0.65, 3.66)		1.67(0.95, 2.95)

*ERAS, Enhanced recovery after surgery; TRAS, Traditional recovery after surgery; RR, Relative risk; CI: confidence interval; DIEP, Deep inferior epigastric artery perforator; MS, Muscle-sparing; TRAM, Transverse rectus abdominis myocutaneous; NA, Not applicable*.

**Table 3:** The meta-analysis results of PCA usage and duration, intravenous injection, and oral morphine consumption; postoperative pain scores; and antiemetic consumption.

**Outcomes**		**Number**	**ERAS**	**TRAS**	**RR/MD, 95%Cl**	**P for RR/MD**	***I*^**2**^**	**P for *I*^**2**^**
Use of PCA		3	22	147	0.17 [0.09, 0.30]	<0.00001	56%	0.1
PCA duration		3	22	147	−10.56 [−20.14, −0.99]	0.03	76%	0.02
Morphine equivalents, IV	POD 0	1	42	49	−1.30 [−2.13, −0.47]	0.002	NA	NA
	POD 1	1	42	49	−11.80 [−13.92, −9.68]	<0.00001	NA	NA
	POD 2	1	42	49	−7.30 [−8.62, −5.98]	<0.00001	NA	NA
	POD 3	1	42	49	−0.50 [−1.75, 0.75]	0.43	NA	NA
	POD 4	1	42	49	1.20 [0.40, 2.00]	0.003	NA	NA
	POD 0–3	1	72	169	−99.00 [−117.56, −80.44]	<0.00001	NA	NA
	Total	2	61	96	−14.87 [−47.36, 17.62]	0.37	91%	0.0006
Morphine equivalents, Oral	POD 0	1	50	50	−35.30 [−54.09, −16.51]	0.0002	NA	NA
	POD 1	2	99	101	−141.01 [−239.39, −42.63]	0.005	89%	0.002
	POD 2	2	99	101	−97.64 [−171.24, −24.05]	0.009	86%	0.007
	POD 3	2	99	101	−50.03 [−90.29, −9.77]	0.01	77%	0.04
	POD 4	1	50	50	−14.00 [−21.41, −6.59]	0.0002	NA	NA
	POD 5	1	50	50	−2.60 [−9.30, 4.10]	0.45	NA	NA
	Total	2	99	101	−307.85 [−486.14, −129.57]	0.0007	84%	0.01
Postoperative pain scores	POD 4 h	2	91	100	−0.15 [−1.62, 1.32]	0.84	0.002	0.02
	POD 8 h	2	91	100	−0.26 [−0.86, 0.35]	0.4	0.007	0.2
	POD 12 h	2	91	100	−0.01 [−0.79, 0.77]	0.98	0.04	0.18
	POD 18 h	2	91	100	0.06 [−0.82, 0.95]	0.89	0.002	0.11
	POD 24 h	2	91	100	0.54 [−2.10, 3.19]	0.69	0.007	<0.00001
	POD 48 h	2	91	100	0.30 [−0.68, 1.28]	0.55	0.04	0.06
	POD 72 h	2	91	100	0.72 [−0.16, 1.60]	0.11	0.002	0.06
	POD 0	1	72	169	−1.10 [−1.54, −0.66]	<0.00001	NA	NA
	POD 0-3	1	72	169	−0.70 [−1.09, −0.31]	0.0004	NA	NA
Antiemetics		3	98	215	0.24 [0.15, 0.37]	0.69	98%	<0.00001

*ERAS, Enhanced recovery after surgery; TRAS, Traditional recovery after surgery; RR, Relative risk; CI: confidence interval; POD, Postoperative day; MD, Mean difference; PCA, Patient-controlled analgesia; IV, Intravenous injection; NA, Not applicable*.

**Figure 5:**
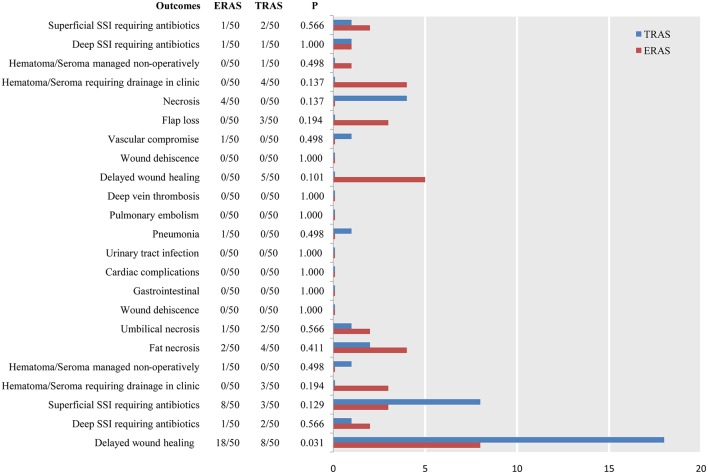
Pooled estimate of the effect of ERAS programs on incidence of breast-related, donor-site, and systemic complications within 45 days after autologous breast reconstruction surgery compared to conventional perioperative care programs.

Only one study (3) reported 45-day postoperative complications. The three most common complications in the ERAS/FTS groups were delayed wound healing at the donor site and breast; and hematoma or seroma at the breast requiring drainage in the clinic. Those in the conventional group were delayed wound healing at the donor site; superficial surgical site infection (SSI) requiring antibiotics at the donor site; and necrosis related to the breast (Figure 5).

#### Complications After Implant-Based Breast Reconstruction Surgery

There was no significant differences between the ERAS/FTS and conventional programs in major (Figure 2; RR, 1.48; 95% CI, 0.60–3.67; *I*^2^ = 18%), minor (Figure 2; RR, 0.68; 95% CI, 0.30–1.53; *I*^2^ = 64%), and breast-related complications (Supplemental Figure 2) at POD 30.

#### Pain Control

Five studies (1, 5, 6, 8, 19) reported the usage rate of analgesics after autologous breast reconstruction surgery. ERAS/FTS was associated with a reduced patient-controlled analgesia (PCA) usage rate (Table 3; RR, 0.17; 95% CI, 0.09–0.30; *I*^2^ = 56%) compared to conventional programs, but there was no significant intergroup difference in PCA duration (Table 3; MD, −10.56; 95% CI, −20.4 to −0.99; *I*^2^ = 76%]. Pooling of the available data revealed that the ERAS/FTS-treated patients had significantly lower postoperative morphine consumption (Table 3).

### Emergency Department Visits, Hospital Readmission, and Unplanned Reoperation

#### Rate After Autologous Breast Reconstruction Surgery

There was no significant difference between the ERAS/FTS and conventional groups in terms of the incidence of hospital readmission (RR, 1.69; 95% CI, 0.99–2.88; *I*^2^ = 0%) or unplanned reoperation (RR, 1.02; 95% CI, 0.30–3.44; *I*^2^ = 42%), within 30 days after surgery (Supplemental Figure 3).

Only one study (5) reported this data within 45 days after surgery. No significant difference between ERAS and conventional programs was noted.

#### Rate After Autologous Breast Reconstruction Surgery

There was no significant difference between the ERAS/FTS and conventional groups in the incidence of hospital readmission or emergency department visits (RR, 0.60; 95% CI, 0.27–1.31; *I*^2^ = 0%] within 30 days after surgery (Supplemental Figure 3).

#### Length of Stay

Eight studies reported LOS in autologous breast reconstruction surgery; of them, two were excluded because the LOS was not defined and contacting the writer was fruitless. Therefore, a total of six studies (1, 5, 6, 8, 19, 20) were included. Pooling of the available data revealed that patients managed with a perioperative ERAS program had mean LOS values that were 1.35-days shorter from admission to discharge (MD, −1.35; 95% CI, −1.75 to −0.95; *I*^2^ = 83.1%), 0.04-days shorter from post-anesthesia care to discharge, and 1.7-nights shorter from admission to discharge than patients in the conventional program (Supplemental Figure 4).

#### Costs

Hospital costs in autologous breast reconstruction surgery were only reported by Oh et al. (24), who considered mean predicted costs and classifications according to Berenson-Eggers Type of Service components (Supplemental Figure 5).

#### Sensitivity Analysis

To explore these results, we performed a stratified analysis across the study strategies. After the exclusion of the case-control study, ERAS/FTS was found to be associated with a statistically significant reduction in the incidence of breast-related infection (RR, 2.18; 95% CI, 1.11–4.27; *I*^2^ = 0%) within 30 days after autologous breast reconstruction surgery. However, there was no significant change in the incidence of breast hematoma or seroma, donor-site infections, LOS (admission to discharge), pneumonia, and urinary tract infection within 30 days after autologous breast reconstruction surgery.

## Discussion

Two other recent reviews compared ERAS/FTS with conventional programs in patients undergoing autologous breast reconstruction surgery. However, Gnaneswaran et al. (11) only included three studies, an inadequate number, and only four outcome measures, which was insufficient to assess the safety and effectiveness of the ERAS program for breast reconstruction surgery. Offodile et al. (13) included six observational studies, three-fifths the number of studies our review included. Moreover, Offodile et al. (13) did not report the implementation of ERAS elements in standard perioperative care program; however, it cannot be ignored that it will definitely weaken the effect of the ERAS program in patients undergoing breast reconstruction surgery. In addition, some details were unreasonable, for instance, the meta-analysis of LOS was based on different units of measurement, while the meta-analysis of complications included complications at POD 30 and 45, which inevitably leads to increasing heterogeneity in the statistical analysis. As a result, further research is necessary.

### Complications

#### Complications After Autologous Breast Reconstruction Surgery

It cannot be ignored that most studies included in the meta-analysis reported higher flap loss rates in the ERAS protocols. However, results that lack significant differences may be attributed to three reasons. Initially, the great majority of ERAS/FTS protocols employed in the review of flap loss within 30 days after surgery, reported the implementation of venous thromboembolism prophylaxis, perioperative intravenous fluid management, early feeding, postoperative flap monitoring, postoperative wound management, and early mobilization, but preadmission optimization, perforator flap planning, and prevention of intraoperative hypothermia were not reported in any studies. Moreover, an insufficient number of studies were included to support the analysis, making the results unstable, and inaccurate. Finally, the definitions of partial and total flap loss and flap type varied.

The American Society of Anesthesiologists physical status scores (25, 26), reconstruction timing and type (27, 28), and age (29–31) at surgery were potentially associated with the incidence of complications. Further research, including studies using the best practices of ERAS program elements as well as exploring the effects of patients' characteristics and different flap types on the incidence of complications, is needed (32). Additionally, some ERAS/FTS elements have been incorporated in conventional programs, which weakens the impact of an ERAS/FTS program to a certain extent, and the definition of major and minor complications and partial and total flap loss will affect the results of the meta-analysis.

#### Complications After Implant-Based Breast Reconstruction Surgery

Some ERAS/FTS elements have been incorporated in conventional programs. Dumestre et al. (21) reported a higher incidence of breast hematoma/seroma in an ERAS program, which may be because some ERAS/FTS elements, including perioperative fasting, antimicrobial prophylaxis, preoperative and intraoperative analgesia, perioperative intravenous fluid management, and postoperative analgesia, were only performed by Dumestre et al. (22). Unfortunately, due to the different total number and types of complications at POD 30 between autologous and implant-based breast reconstruction surgery, comparability was impossible. In addition, although our meta-analysis found a decreased breast-related infection rate with the ERAS protocol, the interpretation of this finding should be considered cautiously because of the larger weight demonstrated by Bonde et al. (20) caused by a large sample size and a limited number of studies.

Most importantly, a prolonged indwelling urinary catheter placement might be associated with urinary tract infections following breast reconstruction surgery. The reason for our meta-analysis result of urinary tract infections may be that only two studies (8, 20) were included in the meta-analysis and the evidence was less robust. Although the relative contribution of each of the single elements in the ERAS/FTS program remains uncertain (32); solid evidence indicated that prolonged indwelling urinary catheter placement can increase the incidence of urinary tract infections (33–35). Removing the urinary catheter on POD 1 is the best practice in ERAS methods.

#### Pain Control

The key factors that keep patients in the hospital after surgery include the need for parenteral analgesia, need for intravenous fluids secondary to gut dysfunction, and bed rest owing to a lack of mobility (36). In addition, pain is an important predictor of postoperative quality of recovery and patient satisfaction. Accordingly, postoperative pain control is essential for early recovery. All studies employed in this review used better practices of venous thromboembolism prophylaxis, preoperative and intraoperative analgesia, perioperative intravenous fluid management, postoperative analgesia, postoperative flap monitoring, and early mobilization, but only Batdorf et al. (8) reported the practice of a standard anesthetic protocol. Surprisingly, ERAS elements were implemented in conventional programs by Kaoutzanis et al. (5), Afonso et al. (6), Batdorf et al. (8), and Odom et al. (23), which weakens the impact of an ERAS/FTS program to a certain extent. Undeniably, the result was not robust owing to the small number of studies included.

#### LOS, Emergency Department Visits, Hospital Readmission, Unplanned Reoperation, and Costs

Most ERAS/FTS protocols employed in the meta-analysis implemented perioperative fasting, preoperative and intraoperative analgesia, perioperative intravenous fluid management, postoperative analgesia, early feeding, postoperative flap monitoring, and early mobilization. Our meta-analysis results showed that the ERAS program shortened preoperative time to a greater extent. Our review showed that LOS may be related to the number of ERAS elements implemented (6, 8, 19, 20). Therefore, setting strict discharge criteria is also essential in minimizing LOS (37). Furthermore, even if a patient met the predefined discharge criteria, hospital discharge might have been delayed for social reasons (38).

A major concern regarding FTS programs is that reduction of the primary hospital stay might result in an increased readmission rate (24, 37). Intriguingly, our meta-analysis showed a strong trend toward a higher readmission rate within 30 days after autologous breast reconstruction surgery treated with the ERAS/FTS program. All four studies showed a higher incidence of hospital readmission in the ERAS/FTS program but did not provide post-discharge home support and physiotherapy. All studies included in the meta-analysis of emergency department visits and unplanned reoperations reported that different degrees of ERAS elements were implemented in conventional programs, which may weaken the difference between ERAS and conventional programs. Moreover, only Kaoutzanis et al. (5) reported these data on POD 45, so the evidence was not robust.

Our review showed that a LOS reduction was associated with lower hospital costs. Postoperative clinical variables, including laterality, hospital readmission, complications, and the need for postoperative blood transfusion had a statistically significant effect on costs reported by Oh et al. (24) only. Further research including multiple studies on cost is needed.

An ERAS program requires a dedicated and motivated team consisting of an anesthesiologist, surgeon, dietician, physiotherapist, social worker, and nursing team (37). Independent programs to reduce harm are not ideal, and it is unlikely that the improved value of surgical care, a hallmark of ERAS, can be accomplished without this transdisciplinary teamwork and coordination. This bundled approach not only serves to bring the team together but also promotes broad implementation of established best-practice principles in concert rather than one at a time (7). By comparing the meta-analysis results and the first but latest consensus in 2017 (10), our research confirmed that the practices of preadmission optimization, perforator flap planning, preventing intraoperative hypothermia perioperative intravenous fluid management (39, 40), and postoperative flap monitoring (20) were associated with a reduced flap loss rate. The practice of preadmission optimization, perforator flap planning, venous thromboembolism prophylaxis, antimicrobial prophylaxis, and intraoperative hypothermia prevention might lead to fewer complications. In addition, the combined practice of perioperative fasting, preoperative, and intraoperative analgesia, perioperative intravenous fluid management, postoperative analgesia, early feeding, postoperative flap monitoring, and early mobilization resulted in a reduced LOS. Our research showed that the combination of venous thromboembolism prophylaxis, preoperative and intraoperative analgesia, perioperative intravenous fluid management, postoperative analgesia, postoperative flap monitoring, and early mobilization led to a decrease in morphine equivalent dosing. However, we could not prove a correlation between the standard anesthetic protocol and less morphine use. An important finding is that early removal of the urinary catheter is presumably associated with fewer urinary tract infections, which is a suggested practice in ERAS treatment.

There are several important limitations to our review. First, in addition to differences in the particular elements that were included in each ERAS program, the number of elements also varied, which created great heterogeneity. ERAS elements were applied in conventional programs. Second, the practices of prophylaxis against venous thromboembolism and the use of preoperative, intraoperative, and postoperative analgesia may result in a higher bleeding risk. Patient satisfaction is critical to the widespread clinical practice of ERAS programs. Owing to only one study (22) demonstrating patient feedback but no relevant data, further studies are needed that report on the amount of bleeding and the degree of patient satisfaction.

## Conclusion

Our study found that the ERAS/FTS program was associated with a significant reduction in morphine consumption and LOS compared to conventional programs. However, there was a trend of higher flap loss rates in the ERAS/FTS-treated patients. In addition, decreased LOS may be associated with higher readmission rates. Most importantly, there is a new insight that removing the urinary catheter on POD 1 is a suggested practice in ERAS programs. The implementation of a comprehensive transdisciplinary program promotes patients to quick postoperative recovery. Additionally, there are several risks of harm. ERAS programs in breast reconstruction should be further confirmed and refined with multicenter prospective randomized trials.

## Data Availability

No datasets were generated or analyzed for this study.

## Author Contributions

G-LG had full access to all of the data in the study and took responsibility for the integrity of the data and accuracy of the data analysis. All authors critically revised the manuscript. G-LG had guarantor.

### Conflict of Interest Statement

The authors declare that the research was conducted in the absence of any commercial or financial relationships that could be construed as a potential conflict of interest.
