# Supplementary material for: Enhanced Recovery After Surgery for Breast Reconstruction: Pooled Meta-Analysis of 10 Observational Studies Involving 1,838 Patients
Source: Front Oncol. 2019 Jul 30;9:675. doi: 10.3389/fonc.2019.00675 (PMC6682620; doi:10.3389/fonc.2019.00675)

**Legends of Supplementary Material**

**Supplemental Method 1** Search strategy

**Supplemental Table 1** Studies excluded from the remaining full texts and the specific reasons for exclusion

**Supplemental Table 2** The number of ERAS elements comparing ERAS to traditional programs

**Supplemental Table 3** Quality assessment of included studies using NOS

**Supplemental Figure 1** Pooled estimate of the effect of ERAS programs on incidence of breast-related complications within 30 days after implant-based breast reconstruction surgery compared to conventional perioperative care programs

**Supplemental Figure 2** Pooled estimate of the effect of ERAS programs on incidence of donor-site complications within 30 days after autologous breast reconstruction surgery compared to conventional perioperative care programs

**Supplemental Figure 3** Pooled estimate of the effect of ERAS programs on incidence of emergency department visits, hospital readmission and unplanned reoperation within 30 days after autologous or implant-based breast reconstruction surgery compared to conventional perioperative care programs

**Supplemental Figure 4** LOS from admission to discharge (days and nights) and from post-anesthesia care unit to discharge (days) in autologous breast reconstruction surgery

**Supplemental Figure 5** Pooled estimate of the effect of ERAS programs on costs and classifications according to Berenson-Eggers Type of Service (BETOS) components compared to conventional perioperative care programs

**Supplemental Method 1 Search strategy**

**1. Ovid MEDLINE(R) Epub Ahead of Print, In-Process & Other Non-Indexed Citations, Ovid MEDLINE(R) Daily, Ovid MEDLINE and Versions(R), 1946 to May 5, 2018**

1. (Enhanced Recovery After Surgery).mp.
2. (Enhanced Recovery Pathways).mp.
3. (ERAS).mp.
4. (Fast-track surgery).mp.
5. (FTS).mp.
6. (Clinical pathway).mp.
7. (Critical pathway).mp.
8. (Multimodal perioperative care).mp.
9. (Accelerated rehabilitation).mp.
10. (Perioperative care).mp.
11. #1 or #2 or #3 or #4 or #5 or #6 or #7 or #8 or #9 or #10
12. exp Mastectomy
13. (Breast reconstruction).mp.
14. (Autologous breast reconstruction).mp.
15. (Breast implants).mp.
16. (Breast surgery).mp.
17. (Free flap surgery).mp.
18. (Reconstructive surgery).mp.
19. (Breast cancer).mp.
20. exp Breast neoplasm
21. (Breast carcinoma).mp.
22. (Acellular dermal matri).ti,ab.
23. Expander.ti,ab.
24. Flap.ti,ab.
25. Graft.ti,ab.
26. Mesh.ti,ab.
27. #12 or #13 or #14 or #15 or #16 or #17 or #18 or #19 or #20 or #21 or #22 or #23 or #24 or #25 or #26
28. #11 and #27

**2. Ovid Embase Classic+Embase, 1947 to May 5, 2018**

1. (Enhanced Recovery After Surgery).mp.
2. (Enhanced Recovery Pathways).mp.
3. (ERAS).mp.
4. (Fast-track surgery).mp.
5. (FTS).mp.
6. (Clinical pathway).mp.
7. (Critical pathway).mp.
8. (Multimodal perioperative care).mp.
9. (Accelerated rehabilitation).mp.
10. (Perioperative care).mp.
11. #1 or #2 or #3 or #4 or #5 or #6 or #7 or #8 or #9 or #10
12. exp Mastectomy
13. (Breast reconstruction).mp.
14. (Autologous breast reconstruction).mp.
15. (Breast implants).mp.
16. (Breast surgery).mp.
17. (Free flap surgery).mp.
18. (Reconstructive surgery).mp.
19. (Breast cancer).mp.
20. exp Breast neoplasm
21. (Breast carcinoma).mp.
22. (Acellular dermal matri).ti,ab.
23. Expander.ti,ab.
24. Flap.ti,ab.
25. Graft.ti,ab.
26. Mesh.ti,ab.
27. #12 or #13 or #14 or #15 or #16 or #17 or #18 or #19 or #20 or #21 or #22 or #23 or #24 or #25 or #26
28. #11 and #27

**3. CENTRAL, The Cochrane Library, 1947 to May 05, 2018**

1. Enhanced Recovery After Surgery
2. Enhanced Recovery Pathways
3. ERAS
4. Fast-track surgery
5. FTS
6. MeSH descriptor: [Clinical pathway] explode all trees
7. Critical pathway
8. Multimodal perioperative care
9. Accelerated rehabilitation
10. Perioperative care
11. #1 or #2 or #3 or #4 or #5 or #6 or #7 or #8 or #9 or #10
12. MeSH descriptor: [Mastectomy] explode all trees
13. Breast reconstruction
14. (Autologous breast reconstruction).mp.
15. (Breast implants).mp.
16. (Breast surgery).mp.
17. (Free flap surgery).mp.
18. (Reconstructive surgery).mp.
19. (Breast cancer).mp.
20. MeSH descriptor: [Breast neoplasm] explode all trees
21. (Breast carcinoma).mp.
22. (Acellular dermal matri):ti,ab,kw.
23. Expander.ti,ab,kw.
24. Flap.ti,ab,kw.
25. Graft.ti,ab,kw.
26. Mesh.ti,ab,kw.
27. #12 or #13 or #14 or #15 or #16 or #17 or #18 or #19 or #20 or #21 or #22 or #23 or #24 or #25 or #26
28. #11 and #27

**Supplemental Table 1. Studies excluded from the remaining full texts and the specific reasons for exclusion**

| **Study** | **Year** | **Title** | **Reasons** |
| --- | --- | --- | --- |
| Momena | 2018 | Outcome of elective caesarean sections following introduction of enhanced recovery programme in obstetrics surgery (EROS) at Whipps Cross University Hospital, Barts Health NHS Trust London, UK | The study design wasn't meet the inclusion criteria. |
| Pihlmann | 2018 | Mastectomy without surgical drainage is safe and feasible | The study design wasn't meet the inclusion criteria. |
| Maciel-Miranda | 2018 | Eclectic breast reconstruction: A tailored approach | The study design wasn't meet the inclusion criteria. |
| lewin | 2018 | Objective evaluation of nipple position after 336 breast reductions | The outcome wasn't meet the inclusion criteria. |
| Kumar | 2018 | PIONEER-Pre-operative Window study of letrozole plus Progesterone receptor agonist Megestrol Acetate versus letrozole alone in post-menopausal patients with OEstrogen Receptor-positive breast cancer | The study design wasn't meet the inclusion criteria. |
| Kim | 2018 | Effect of goal-directed haemodynamic therapy in free flap reconstruction for head and neck cancer | The study design wasn't meet the inclusion criteria. |
| Ikink | 2018 | Insertional mutagenesis in a HER2-positive breast cancer model reveals ERAS as a driver of cancer and therapy resistance | The study design wasn't meet the inclusion criteria. |
| Chiu | 2018 | Improved analgesia and reduced post-operative nausea and vomiting after implementation of an enhanced recovery after surgery (ERAS) pathway for total mastectomy | The study design wasn't meet the inclusion criteria. |
| Temple-Oberle | 2017 | Consensus Review of Optimal Perioperative Care in Breast Reconstruction: Enhanced Recovery after Surgery (ERAS) Society Recommendations | The study design wasn't meet the inclusion criteria. |
| Spicka | 2017 | Influence of Enhanced Recovery Regime on Early Outcomes of Total Knee Arthroplasty | The study design wasn't meet the inclusion criteria. |
| Sebai | 2017 | Enhanced recovery after surgery pathway for microsurgical breast reconstruction: a systematic review and meta-analysis | The study design wasn't meet the inclusion criteria. |
| Moran | 2017 | Tissue expansion for breast reconstruction: Methods and techniques | The outcome wasn't meet the inclusion criteria. |
| Lin | 2017 | Discussion: Consensus Review of Optimal Perioperative Care in Breast Reconstruction: Enhanced Recovery after Surgery (ERAS) Society Recommendations | The study design wasn't meet the inclusion criteria. |
| Latt | 2017 | Impact of hepatitis C treatment on survival outcomes among liver transplant recipients with recurrent infection in the era of direct-acting anti-viral agents | The study design wasn't meet the inclusion criteria. |
| Anolik | 2017 | Enhanced Recovery after Surgery (ERAS) implementation and multimodal pain management in abdominal based free flap breast reconstruction | The study design wasn't meet the inclusion criteria. |
| Kontopodis | 2017 | Intraoperative Techniques for the Plastic Surgeon to Improve Pain Control in Breast Surgery | The study design wasn't meet the inclusion criteria. |
| Harvey | 2017 | Local anaesthetic wound catheter use after implant based breast reconstruction e Towards enhanced recovery | The study design wasn't meet the inclusion criteria. |
| Lange | 2016 | Chronic Periprosthetic Hip Joint Infection. A Retrospective, Observational Study on the Treatment Strategy and Prognosis in 130 Non-Selected Patients | The study design wasn't meet the inclusion criteria. |
| Hayes | 2016 | Audit of hospital recovery and complications of breast reconstruction patients: An overview for implementing an enhanced recovery protocol-pilot study | The study design wasn't meet the inclusion criteria. |
| Garin | 2016 | Natural hydroxyapatite as a bone graft extender for posterolateral spine arthrodesis | The study design wasn't meet the inclusion criteria. |
| Armstrong | 2016 | Determinants of increased acute postoperative pain after autologous breast reconstruction within an enhanced recovery after surgery protocol: A prospective cohort study | The outcome wasn't meet the inclusion criteria. |
| Afonso | 2016 | ERAS implementation in breast reconstructive surgery: Which elements are important? | The study design wasn't meet the inclusion criteria. |
| Hojvig | 2015 | Breast reconstruction with m. latissimus dorsi--status and perspectives | The study design wasn't meet the inclusion criteria. |
| Donald | 2015 | Optimising the enhanced recovery programme at Royal Bolton Hospital for non-reconstructive breast surgery | The study design wasn't meet the inclusion criteria. |
| Dhanancheyan | 2015 | Survey of UK anaesthetists on use of enhanced recovery for mastectomy | The study design wasn't meet the inclusion criteria. |
| Al Omran | 2015 | Re: 'Enhanced recovery after surgery in microvascular breast reconstruction' | The outcome wasn't meet the inclusion criteria. |
| Watts | 2015 | Introduction of enhanced recovery after surgery (ERAS) pathways in latissimus dorsi flap reconstruction | The study design wasn't meet the inclusion criteria. |
| Sadideen | 2014 | The national variation in peri-operative anaesthetic technique for breast free flap reconstruction in the UK: is it time to define best practice? | The study design wasn't meet the inclusion criteria. |
| Das | 2014 | Xenogenic transplantation of human breast adipose-derived stromal vascular fraction enhances recovery of erectile function in diabetic mice | The study design wasn't meet the inclusion criteria. |
| Ribuffo | 2014 | Treatment of irradiated expanders: protective lipofilling allows immediate prosthetic breast reconstruction in the setting of postoperative radiotherapy | The study design wasn't meet the inclusion criteria. |
| Mertz | 2013 | Fast-track surgery for breast cancer is possible | The study design wasn't meet the inclusion criteria. |
| Jordan | 2013 | Use of the Enhanced Recovery After Surgery (ERAS) programme in an onco-plastic surgical setting | The study design wasn't meet the inclusion criteria. |
| Pronisceva | 2012 | Same day discharge after mastectomy and axillary clearance? | The study design wasn't meet the inclusion criteria. |
| Ohara | 2012 | Effects of a preoperative carbohydrate-rich drink on postoperative nausea and vomiting after mastectomy | The study design wasn't meet the inclusion criteria. |
| Carruthers | 2012 | Comprehensive breast enhanced recovery programme facilitates early discharge | The study design wasn't meet the inclusion criteria. |
| Ament | 2012 | Sustainability of healthcare innovations (SUSHI): long term effects of two implemented surgical care programmes (protocol) | The study design wasn't meet the inclusion criteria. |
| Arsalani-Zadeh | 2011 | Evidence-based review of enhancing postoperative recovery after breast surgery | The outcome wasn't meet the inclusion criteria. |
| Williams | 2010 | Fast-track surgery and nursing care | The study design wasn't meet the inclusion criteria. |
| Saporito | 2010 | Thoracic paravertebral block as the sole anaesthetic technique for breast surgery: Comparison with a combined anaesthesia | The study design wasn't meet the inclusion criteria. |
| Peters | 2009 | Complications from injectable materials used for breast augmentation | The outcome wasn't meet the inclusion criteria. |

**Supplemental Digital Table 2.** Number of ERAS elements compared to conventional programs

| Study | I | Enhanced Recovery After Surgery/Fast-Tract Surgery Interventions | | | | | | | | | | | | | | | | | | Total |
| --- | --- | --- | --- | --- | --- | --- | --- | --- | --- | --- | --- | --- | --- | --- | --- | --- | --- | --- | --- | --- |
|  |  |  |  |  |  |  |  |  |  |  |  |  |  |  |  |  |  |  |  |  |
| Afonso  2017[6] | E = 42 |  |  |  | ✔ |  | ✔^1^ |  | ✔^2^ | ✔^2^ |  |  | ✔ | ✔^3^ | ✔ | ✔^4^ |  | ✔ |  | 9 |
|  | C = 49 |  |  |  |  |  |  |  |  | ✔^2^ |  |  |  |  |  | ✔^4^ |  |  |  | 2 |
| Astanehe  2018[15] | E = 72 |  |  |  | ✔^5^ | ✔^6^ | ✔ | ✔^7^ | ✔ | ✔ |  |  | ✔^8^ | ✔ | ✔ | ✔ | ✔ | ✔ |  | 12 |
|  | C = 169 |  |  |  |  |  |  |  |  |  |  |  |  |  |  |  |  |  |  | 0 |
| Batdorf  2015[7] | E = 49 | ✔ |  |  | ✔ |  | ✔^1^ | ✔ |  | ✔ | ✔ |  | ✔^8^ | ✔ | ✔ | ✔ | ✔ | ✔ |  | 12 |
|  | C = 51 |  |  |  |  |  | ✔^9^ | ✔^9^ |  |  |  |  |  |  |  |  |  |  |  | 2 |
| Bonde  2015[16] | E = 177 | ✔ |  |  |  |  |  |  |  |  | ✔ |  |  | ✔ |  | ✔^10^ | ✔ | ✔ |  | 6 |
|  | C = 277 |  |  |  |  |  |  |  |  |  | ✔ |  |  | ✔^11^ |  | ✔ | ✔ |  |  | 4 |
| Chiu  2018[1] | E = 96 | ✔ |  |  | ✔ |  |  |  | ✔^12^ | ✔ | ✔ | ✔ | ✔^2^ | ✔ | ✔^13^ |  |  | ✔^13^ |  | 10 |
|  | C = 276 |  |  |  |  |  |  |  |  |  |  |  |  |  |  |  |  |  |  | 0 |
| Dumestre  2017a[17] | E = 78 | ✔ | ✔^14^ |  |  |  |  |  | ✔ |  |  |  |  |  |  |  |  |  | ✔ | 4 |
|  | C = 78 |  | ✔^14^ |  |  |  |  |  |  |  |  |  |  |  |  |  |  |  |  | 1 |
| Dumestre  2017b[18] | E = 29 | ✔ | ✔^14^ |  | ✔^5^ |  |  | ✔^15^ | ✔ | ✔ |  |  | ✔^2^ | ✔ |  |  |  |  | ✔ | 9 |
|  | C = 29 |  | ✔^14^ |  |  |  |  |  |  |  |  |  |  |  |  |  |  |  |  | 1 |
| Kaoutzanis  2018[3] | E = 50 | ✔ |  |  | ✔ |  | ✔ | ✔^15^ | ✔ | ✔ |  |  | ✔^8^ | ✔ | ✔ | ✔^4^ |  | ✔ |  | 11 |
|  | C = 50 |  |  |  | ✔^16^ |  | ✔^17^ | ✔ | ✔^18^ |  |  |  | ✔^19^ | ✔ | ✔ | ✔^4^ |  | ✔^20^ |  | 9 |
| Oh  2018[19] | E = 82 |  |  |  |  |  |  |  |  | ✔ |  |  | ✔ | ✔ | ✔ | ✔ |  | ✔ |  | 6 |
|  | C = 118 |  |  |  |  |  |  |  |  |  |  |  | ✔ | ✔ |  |  |  |  |  | 2 |
| Odom | E = 19 |  |  |  | ✔^21^ | ✔^21^ | ✔ | ✔^21^ | ✔^22^ | ✔ |  |  | ✔^17^ | ✔ | ✔ | ✔ |  | ✔ |  | 11 |
| 2017[20] | C = 47 |  |  |  |  |  |  |  |  | ✔^1^ |  |  |  |  |  |  |  |  |  | 1 |

**Notes:** Preadmission information, education, and counseling; Preadmission optimization; Perforator flap Planning; Perioperative fasting; Preoperative carbohydrate loading; Venous thromboembolism prophylaxis; Antimicrobial prophylaxis; Postoperative nausea and vomiting prophylaxis; Preoperative and intraoperative analgesia; Standard anesthetic protocol; Preventing intraoperative hypothermia; Perioperative intravenous fluid management; Postoperative analgesia; Early feeding; Postoperative flap monitoring; Postoperative wound management; Early mobilization; Postdischarge home support and physiotherapy; I, intervention; E, Enhanced Recovery After Surgery/Fast-Tract Surgery; C, conventional/traditional perioperative care program; ✔, The practice of this element is consistent with the consensus description; ^1^ Only be implemented in the preoperation; ^2^ Only be implemented in the intraoperation; ^3^ Opioid as one of the analgesics; ^4^ The period and frequency of monitoring are unclear; ^5^ May drink clear fluids up to 3h prior to surgery; ^6^ Carbohydrate-rich juice evening and morning of surgery; ^7^ Intravenous Ancef 2g in the preoperation, intraoperation and postoperation; ^8^ Be implemented in the intraoperation and postoperation; ^9^ The specific time of implementation is unknown; ^10^ The flap-monitoring period is 2 days; ^11^ Be implemented by continuous epidural aenalgesia; ^12^ Be implemented on patients who are younger than the age of 60 years, with a history of PONV; ^13^ Be emphasized after surgery, but the specific time is unknowed; ^14^ American Society of Anesthesiologists class 1 or 2 and a body mass index less than 35 kg/m^2^; ^15^ Intravenous Cefazolin; ^16^ Be kept nil by mouth for six hours prior to surgery; ^17^ Only be implemented in the postoperation; ^18^ Not standardized and includs intravenous opiate analgesics or patient-controlled analgesia(PCA) pump; ^19^ Be used until the patient tolerate an unrestricted diet; ^20^ Be encouraged but not expected; ^21^ The interval between the implemention of this item and surgery is not clear; 22 Be implemented in intraoperation and postoperation.

**Supplemental Digital Table 3.** Quality assessment of included studies, using Newcastle-Ottawa Scale (NOS)

| **Author** | **Year** | **Study design** | **Selection** | | | |  | **Comparability** | |  | **Outcomes** | | | **Total** |
| --- | --- | --- | --- | --- | --- | --- | --- | --- | --- | --- | --- | --- | --- | --- |
|  |  |  | **Representativeness of the exposed cohort** | **Selection of the non exposed cohort** | **Ascertainment of exposure** | **Demonstration that outcome of interest was not present at start of study** |  | **Comparability of cohorts on the basis of the design or analysis** | |  | **Assessment of outcome** | **Was follow-up long enough for outcomes to occur** | **Adequacy of follow up of cohorts** |  |
| Afonso  [6] | 2017 | Cohort study | 0 | 1 | 1 | 1 |  | 1 | 1 |  | 1 | 1 | 1 | **8** |
| Astanehe  [15] | 2018 | Cohort study | 0 | 1 | 1 | 1 |  | 0 | 1 |  | 1 | 1 | 1 | **7** |
| Batdorf  [7] | 2015 | Cohort study | 0 | 0 | 1 | 1 |  | 1 | 1 |  | 1 | 1 | 1 | **7** |
| Chiu  [1] | 2016 | Cohort study | 1 | 1 | 1 | 1 |  | 0 | 1 |  | 1 | 0 | 0 | **6** |
| Dumestre  a[17] | 2017 | Cohort study | 0 | 0 | 1 | 1 |  | 0 | 1 |  | 1 | 1 | 1 | **6** |
| Dumestre  b[18] | 2017 | Cohort study | 0 | 0 | 1 | 1 |  | 0 | 1 |  | 1 | 1 | 1 | **7** |
| Kaoutzanis  [3] | 2018 | Cohort study | 0 | 1 | 1 | 1 |  | 0 | 1 |  | 1 | 1 | 1 | **7** |
| Oh  [19] | 2018 | Cohort study | 0 | 1 | 1 | 1 |  | 0 | 0 |  | 1 | 1 | 1 | **6** |
| Odom | 2017 | Cohort  study | 0 | 1 | 1 | 1 |  | 1 | 1 |  | 1 | 0 | 0 | **6** |
| [20] |  |  |  |  |  |  |  |  |  |  |  |  |  |  |
| **Author** | **Year** | **Study design** | **Is the case definition adequate** | **Representativeness of the cases** | **Selection of controls** | **Definition of controls** |  | **Comparability of cases and controls on the basis of the design or analysis** | |  | **Ascertainment of exposure** | **Same method of ascertainment for cases and controls** | **Non-response rate** | **Total** |
| Bonde[16] | 2015 | Case control study | 1 | 0 | 1 | 1 |  | 0 | 0 |  | 1 | 1 | 1 | **6** |

**Supplemental Figure 1.** Pooled estimate of the effect of ERAS programs on incidence of breast-related complications within 30 days after implant-based breast reconstruction surgery compared to conventional perioperative care programs


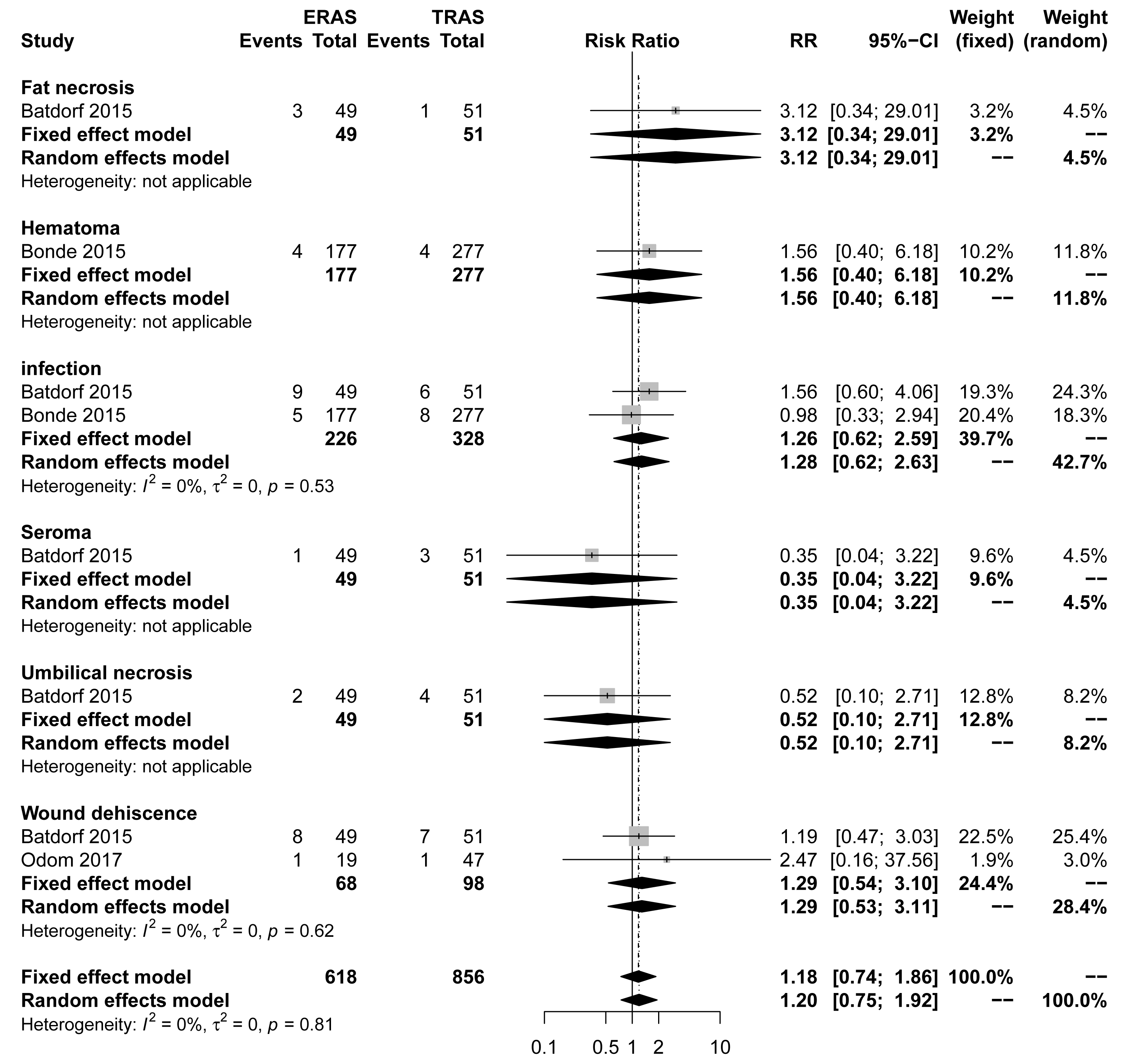


**Supplemental Figure 2.** Pooled estimate of the effect of ERAS programs on incidence of donor-site complications within 30 days after autologous breast reconstruction surgery compared to conventional perioperative care programs

**
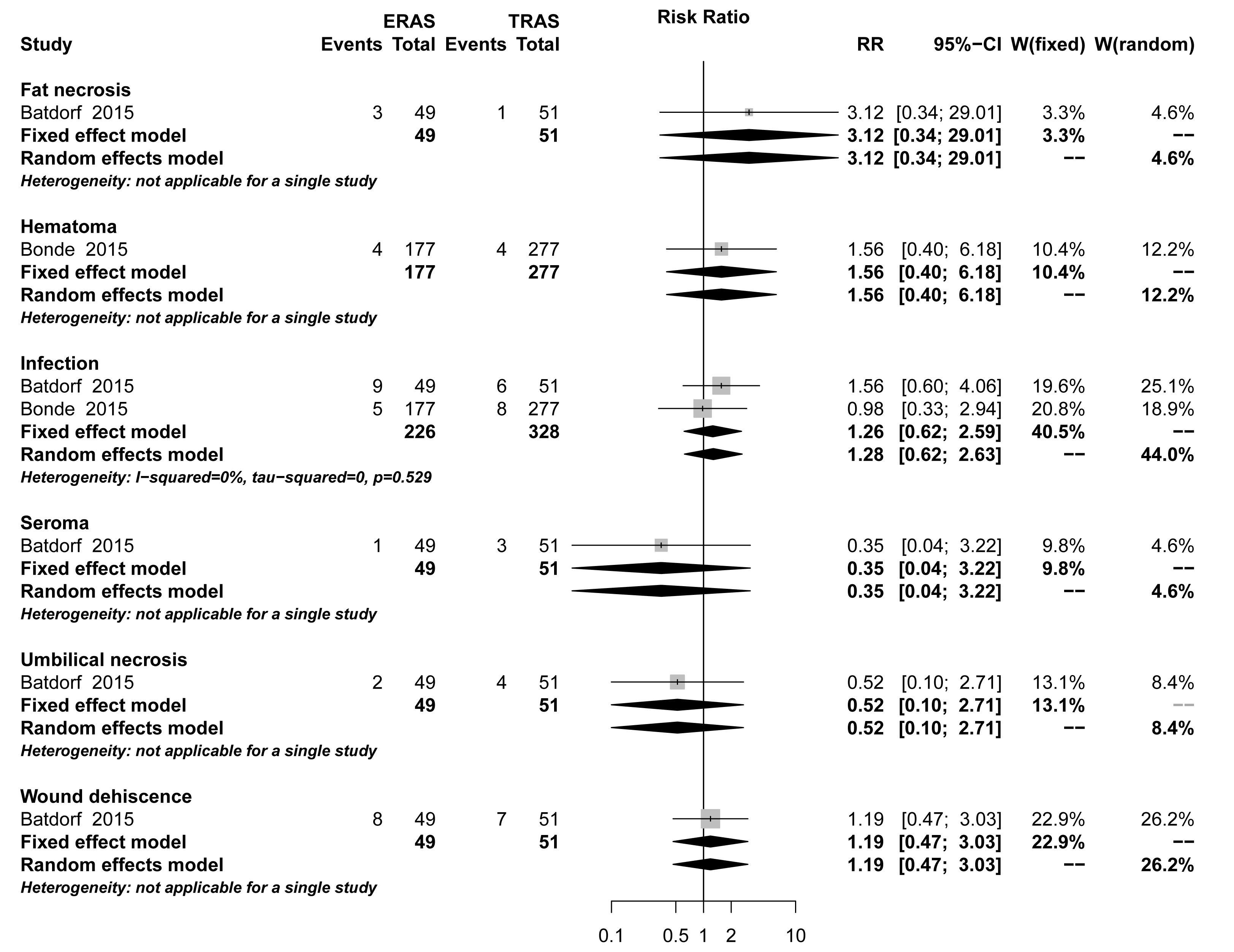
**

**Supplemental Figure 3.** Pooled estimate of the effect of ERAS programs on incidence of emergency department visits, hospital readmission and unplanned reoperation within 30 days after autologous or implant-based breast reconstruction surgery compared to conventional perioperative care programs

**
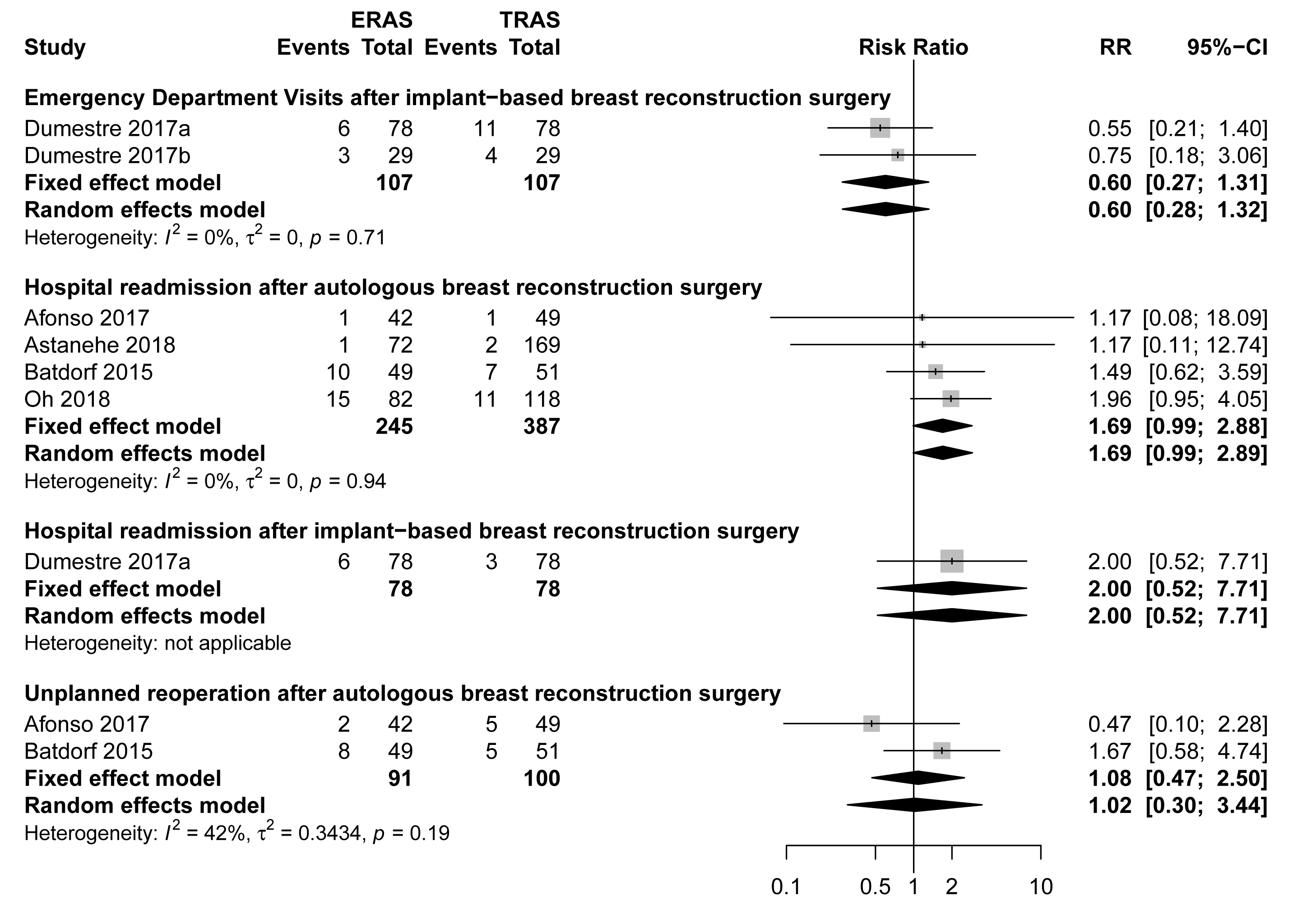
**

**Supplemental Figure 4.** LOS from admission to discharge (days and nights) and from post-anesthesia care unit to discharge (days) in autologous breast reconstruction surgery

**
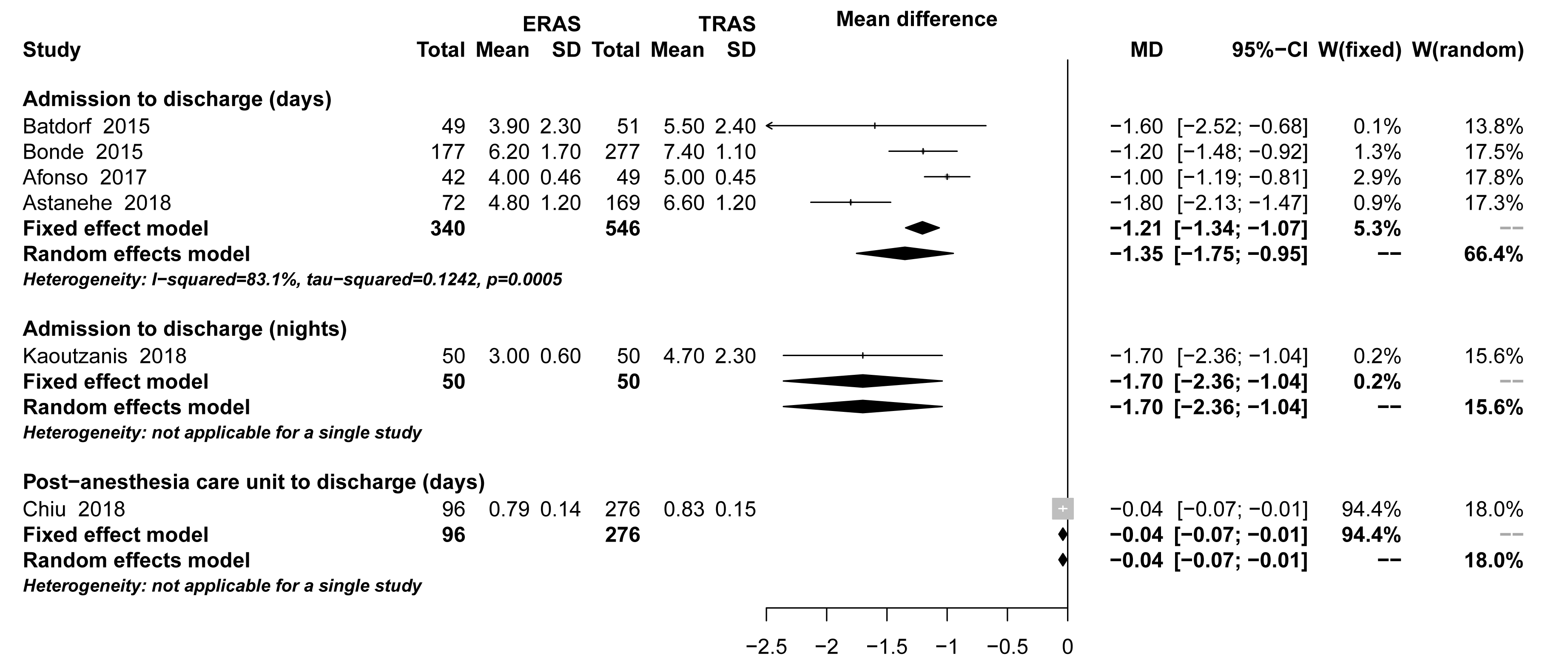
**

**Supplemental Figure 5.** Pooled estimate of the effect of ERAS programs on costs and classificationsaccording to Berenson-Eggers Type of Service (BETOS) components compared to conventional perioperative care programs


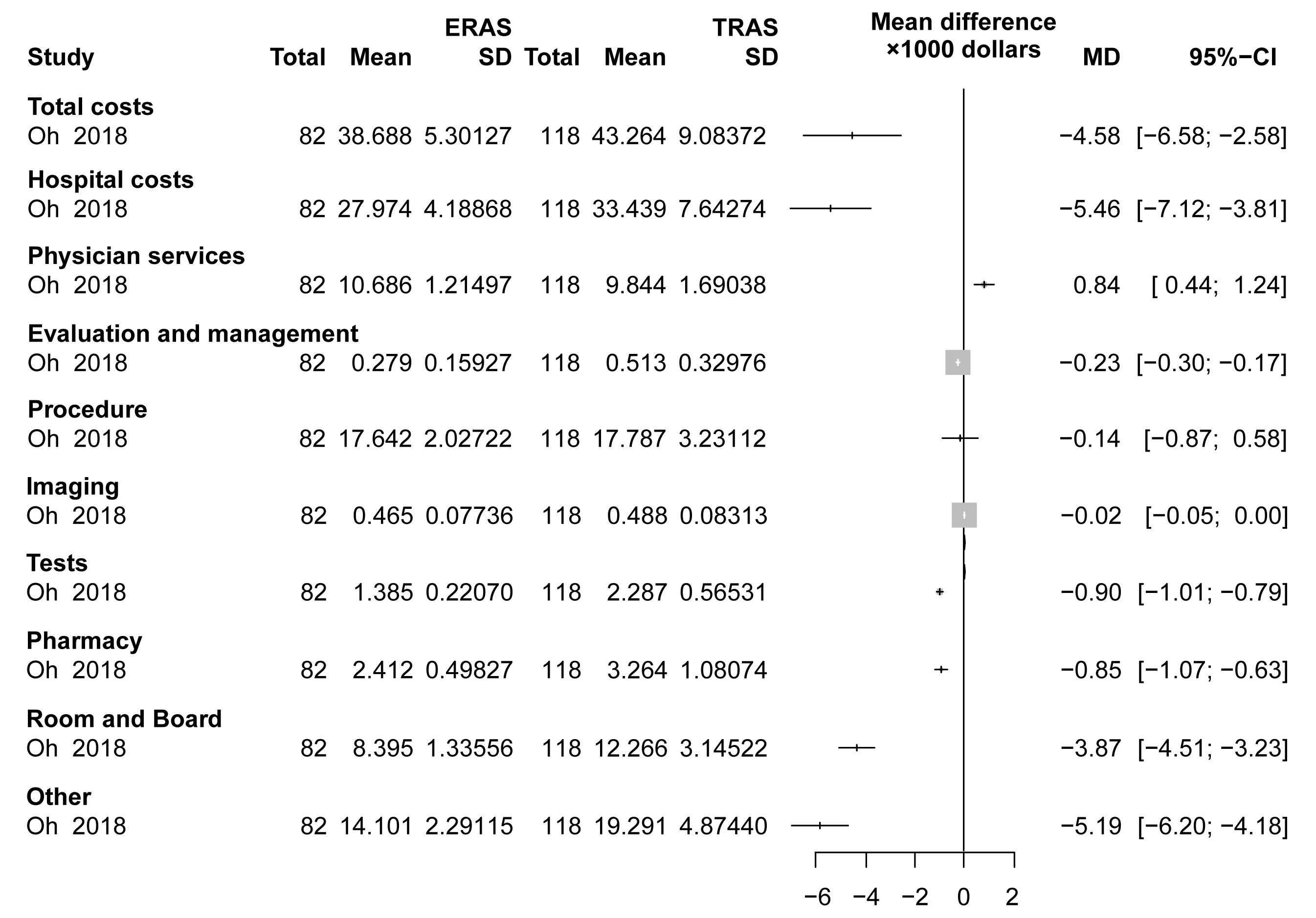

Supplement: Supplementary file 1 [file Table_1.DOC]
